# Pannexin 1 channels: new actors in the regulation of catecholamine release from adrenal chromaffin cells

**DOI:** 10.3389/fncel.2014.00270

**Published:** 2014-09-04

**Authors:** Fanny Momboisse, María José Olivares, Ximena Báez-Matus, María José Guerra, Carolina Flores-Muñoz, Juan C. Sáez, Agustín D. Martínez, Ana M. Cárdenas

**Affiliations:** ^1^Centro Interdisciplinario de Neurociencias de Valparaíso, Universidad de ValparaísoValparaíso, Chile; ^2^Departamento de Fisiología, Pontifícia Universidad Católica de ChileSantiago, Chile

**Keywords:** pannexin 1, Ca^2+^ signaling, neurosecretion, catecholamines, chromaffin cells

## Abstract

Chromaffin cells of the adrenal gland medulla synthesize and store hormones and peptides, which are released into the blood circulation in response to stress. Among them, adrenaline is critical for the fight-or-flight response. This neurosecretory process is highly regulated and depends on cytosolic [Ca^2+^]. By forming channels at the plasma membrane, pannexin-1 (Panx1) is a protein involved in many physiological and pathological processes amplifying ATP release and/or Ca^2+^ signals. Here, we show that Panx1 is expressed in the adrenal gland where it plays a role by regulating the release of catecholamines. In fact, inhibitors of Panx1 channels, such as carbenoxolone (Cbx) and probenecid, reduced the secretory activity induced with the nicotinic agonist 1,1-dimethyl-4-phenyl-piperazinium (DMPP, 50 μM) in whole adrenal glands. A similar inhibitory effect was observed in single chromaffin cells using Cbx or ^10^Panx1 peptide, another Panx1 channel inhibitors. Given that the secretory response depends on cytosolic [Ca^2+^] and Panx1 channels are permeable to Ca^2+^, we studied the possible implication of Panx1 channels in the Ca^2+^ signaling occurring during the secretory process. In support of this possibility, Panx1 channel inhibitors significantly reduced the Ca^2+^ signals evoked by DMPP in single chromaffin cells. However, the Ca^2+^ signals induced by caffeine in the absence of extracellular Ca^2+^ was not affected by Panx1 channel inhibitors, suggesting that this mechanism does not involve Ca^2+^ release from the endoplasmic reticulum. Conversely, Panx1 inhibitors significantly blocked the DMPP-induce dye uptake, supporting the idea that Panx1 forms functional channels at the plasma membrane. These findings indicate that Panx1 channels participate in the control the Ca^2+^ signal that triggers the secretory response of adrenal chromaffin cells. This mechanism could have physiological implications during the response to stress.

## Introduction

The release of hormones from neuroendocrine cells is a highly regulated process that has to be adjusted to environmental demands. This regulation is particularly required for the release of catecholamines during stress situations wherein the body reacts to environmental changes (Harvey et al., 1984). The adrenal gland medulla constitutes the major source of catecholamines in the body (Wong, 2003). These glands are innervated by cholinergic terminals of the splanchnic nerve, which activates chromaffin cells in response to stressors. Upon activation with acetylcholine, chromaffin cells secrete to the blood circulation mostly catecholamines (adrenaline, noradrenaline) but also ATP, enkephalins and neuropeptide Y among others peptides and proteins (Aunis and Langley, 1999; Crivellato et al., 2008). The release process depends on a series of events that are finely regulated and occur in a perfect sequence: activation of nicotinic receptors, membrane depolarization, opening of voltage-dependent Ca^2+^ channels, and transient increase of the cytosolic [Ca^2+^] that ends with the exocytotic release of molecules stored in chromaffin granules (Weiss, 2012). Most of the released transmitters act on autoreceptors present in the chromaffin cell plasma membrane allowing an autocrine feedback of the secretory process (Currie and Fox, 1996; Lim et al., 1997).

Pannexins (Panxs) are a family of three glycoproteins, Panxs 1, 2 and 3, which form pore channels localized at the plasma membrane but also in intracellular compartments such as the endoplasmic reticulum (ER; Penuela et al., 2008). Pannexin-1 (Panx1), the best-characterized member, is involved in various cellular processes in both pathologic and physiologic contexts (Thompson et al., 2008; Orellana et al., 2011; Séror et al., 2011), where it participates in the initiation and/or amplification of ATP release and Ca^2+^ signals (Bao et al., 2004; Vanden Abeele et al., 2006). Most of the described actions of Panx1 channels often take place in tight collaboration with metabotropic or ionotropic purinergic receptors. Furthermore, Panx1 forms a functional association with the purinergic receptor P2X_7_ (P2X_7_R) (Locovei et al., 2007). It has been proposed that these types of associations allow an autocrine feedback loop that controls different physiological settings, such as the immune synapse for T-cell activation (Woehrle et al., 2010) or the potentiation of the skeletal muscle contraction (Riquelme et al., 2013). These evidences place Panx1 as an important actor in the control of several signaling processes, mainly through its participation in the regulation of the cytosolic [Ca^2+^]. Hence, we decided to study the possible role of Panx1 in the process of catecholamine release from the adrenal medulla and its underlying mechanism. We found that Panx1 is expressed at the plasma membrane of adrenal chromaffin cells where it plays an important role controlling the release of catecholamines through the amplification of Ca^2+^ signals triggered by activation of nicotinic receptors.

## Materials and methods

### Chromaffin cell culture

Bovine adrenal chromaffin cells were isolated as previously described (Montiel et al., 2003). Cells were plated at a density of 10 × 10^6^ cells/ml on collagen-coated glass coverslips (12 or 25 mm, Warner Instrument, Hamden, USA) or on plastic culture dishes (10-cm diameter, Orange scientific, Braine-l’Alleud, Belgium), and kept at 37°C, 5% CO_2_ and 100% humidity at least 48 h before the experiments.

### Inhibitors, dye and antibodies

Carbenoxolone (Cbx) and probenecid were purchased from Sigma-Aldrich (Saint Louis, MO, USA) and Molecular Probes Life technologies (Grand Island, USA), respectively. ^10^Panx1 and ^10^Panx1 scramble (^10^Panx1 scrb) peptides were synthesized by Tocris Bioscience (Bristol, UK). A rabbit polyclonal anti-Panx1 serum was a generous gift from Dr. D. Laird (University of Western Ontario, London, Canada). The monoclonal anti-actin antibody and 4′,6-diamidino-2-phenylindole (DAPI) were purchased from Sigma-Aldrich. The α-CY2-labeled secondary anti-rabbit antibody and the HRP-labeled secondary anti-rabbit antibody were purchased from Jackson ImmunoResearch (West Grove, USA) and the HRP-labeled secondary anti-mouse antibody was obtained from Thermoscientific (Waltham, USA) respectively.

### RT-PCR and single cell RT-PCR

#### RT-PCR

Total RNA from bovine adrenal glands or cultured bovine chromaffin cells was isolated using the SV Total RNA Isolation System from Promega (Madison, USA), following the manufacturer’s instructions. cDNAs were synthesized from total RNAs using SUPERSCRIPT III First-Strand (Invitrogen). Hot start PCR was performed using 2 ng cDNA in a total volume of 50 μl containing PCR Master Mix (Promega) and specific primers (forward primer 5′ TACTTTGGGGATGCCTGGAG-3′ and reverse primer 5′-GGCGCACTGAAAGACCTC-3′ for dopamine β-hydroxylase (DBH), matched with gene ID NM_180995.2, targeting exon-exon junction with the forward primer, forward primers 5′-CGCAAGAAATCTCCATTGGT-3′ and reverse primer for Panx1, 5′-GGCTTTCCTGTGAACTTTGC-3′, matched with gene ID: NM_001245925.1, targeting exon-exon junction with the reverse primer and forward primer 5′-TTTGTGATGGGTGTGAACCACGAG-3′ and reverse primer 5′-CAACGGATACATTGGGGGTAGGAAC-3′ for GADPH, matched with the gene ID XR 405643.1. PCR reactions were run for 40 cycles and PCR products obtained for DBH (480 pb), Panx1 (396 pb) and GADPH (334 pb) were visualized in 4% agarose gel.

#### Single cell RT-PCR

The protocol for Single cell RT-PCR was adapted from Phillips and Lipski (2000). Briefly, the cytoplasm of a single cultured cell was picked up using a patch pipette filled with 10 μl of autoclaved diethylpyrocarbonate-treated water. The cell content was expelled by negative pressure into an autoclaved microtube, immediately frozen in liquid nitrogen and keep at −80°C. The reverse transcription was performed as described above. For amplification of the cDNA product, we used a Nested PCR procedure. During the first round of PCR (40 cycles, as described above), Panx1 and DBH external primer pairs (sequence mentioned above) were mixed in the PCR reaction. The 50 μl containing the PCR product was split in two tubes and the second PCR was performed using the internal primers (forward: 5′ TGTGACCCCAACGACTACCT 3′, and reverse primers: 5′ TCGGTCACGTAGCACCAGTA 3′ for DBH, matched with gene ID NM_180995.2, and 5′ TAAGCTGCTTCTCCCCCAGT 3′, and 5′ AGGCACCGTCTCTCAAGTCA 3′ for Panx1, matched with gene ID NM_001245925.1). PCR products (248 pb for DBH and 318 pb for Panx1) were analyzed in 4% agarose gel. For each RT-PCR, negative controls were prepared using water as template.

### Western blot analysis

The presence of Panx1 was evaluated in extracts of bovine adrenal glands, cultured chromaffin cells, wild type or Panx1^−/−^ KO mice brain using western blot analysis. Tissue or cell extracts were obtained using a lysis buffer (HaltTM Protease and Phosphatase Inhibitor Cocktail) (Thermoscientific, Waltham, USA) and 0.5 μM EDTA in phosphate buffered saline (PBS) pH 7.4. After 10 s of sonication, extracts were centrifuged 10 min at 14,000 rpm 4°C. The supernatants were collected and proteins were quantified using Qubit® Protein Assay (Molecular Probes, Life Technologies). The amount of 40 μg of proteins of each extract was separated by electrophoresis on a 10% SDS-polyacrylamide gel and proteins were electrotransferred to nitrocellulose membranes. Then, membranes were incubated for 1 h with blocking solution (0.05% Tween-20 with 5% w/v nonfat dry milk) in a Tris-buffered saline (TBS) solution, followed by overnight incubation at 4°C with anti-Panx1 serum diluted (1:1000) in blocking solution. After several washes, membranes were incubated with HRP-labeled secondary anti-rabbit antibody (1:5000) for 1 h and immunoreactive bands were revealed with the ECL Plus system (Amersham GE Lifes sciences, Piscataway, USA). As Actin and Panx1 have similar molecular weight, the membrane were treated with a stripping buffer (glycine 0.2 M, 0.1% SDS, Tween 20 1%, pH 2.2) and then washed, incubated with the blocking solution as described above and incubated with anti-actin antibody (1/500, 4°C overnight in blocking solution). After several washes membranes were incubated with HRP-labeled secondary anti-mouse antibody (1:2500) for 1 h and immunoreactive bands were revealed as described above.

### Catecholamine secretion from the adrenal gland

Fresh bovine adrenal glands were perfused with a Kreb’s-Hepes solution (in mM: 140 NaCl, 5.9 KCl, 1.2 MgCl_2_, 2 CaCl_2_, 10 Hepes-NaOH, 10 glucose) by means of a peristaltic pump (Variable-speed pump 2, Fisher Scientific, Waltham, USA) at a rate of 4 mL/min. The solution was constantly bubbled with O_2_ and the final pH was maintained in the range of 7.4–7.5. After an equilibration time of 1 h, secretion was induced with a 2 min pulse of the nicotinic agonist 1,1-dimethyl- 4-phenyl-piperazinium (DMPP, 50 μM) added into the perfusion stream. A second pulse was applied 30 min later. In order to evaluate the role of Panx1 channels in the catecholamine release, a group of glands was perfused 15 min before and during the second DMPP pulse with Cbx (5 μM) or probenecid (200 μM).

Perfusates were collected in 2 min fractions, 6 min prior to the DMPP pulse to determine the spontaneous secretion of catecholamines, during the pulse, and 14 min after the DMPP pulse. The perfusated samples were collected in tubes containing 0.005 N perchloric acid and kept on ice. The catecholamines released in the background sample were subtracted from those released from the stimulated sample to obtain the net value of secretion. The products generated from the oxidation of noradrenaline and adrenaline by iodine at pH 6 were measured (excitation wavelength 540 nm) (Persky, 1955). Due to the variation in gland shape and size, each gland was used as its own control, thus results were expressed as the percentage of the first stimulation pulse.

### Immunofluorescence and confocal imaging

Cultured chromaffin cells were first washed with PBS (pH 7.4), and incubated fixed with 4% paraformaldehyde (PFA) in PBS for 15 min at 4°C and permeabilized with a fixative solution containing 0.2% Triton X-100 for 10 min. Then, cells were rinsed several times with PBS, pre-treated with 3% bovine serum albumin (BSA) in PBS for 1 h, and incubated with the anti-Panx1 serum (1:100) for 2 h. After that, cells were washed several times with PBS and incubated for 1 h with α-CY2-labeled secondary anti-rabbit antibody (1:1000). After several washes with PBS, cells were incubated with DAPI (5 μg/ml) for 15 min. Finally, coverslips were washed and mounted with Dako fluorescent mounting medium (Dako, Glostrup, Denmark). Stained cells were visualized with a Nikon C1 Plus laser-scanning confocal microscope, equipped with a 100X objective NA 1.30 and excited with laser line 408 and 488 nm. To determine if Panx1 is localized at the plasma membrane, cultured chromaffin cells were labeled with extracellular biotin. Cells were first washed in PBS (pH 7.4) and incubated 10 min with a solution of Kreb’s Hepes containing 90 μM sulfo-NHS-biotin (Thermoscientific Waltham, USA). Then, cells were incubated twice with 15 mM glycin solution (in Kreb’s Hepes) for 7 min and then fixed with 4% PFA. The immunofluorescence protocol was similar as described above but using an avidin-Cy3 (1:5000, Jackson ImmunoResearch) with the α-CY2-labeled secondary anti-rabbit antibody. Confocal acquisitions were analyzed and processed using Image-J software (NIH, USA). Biotin or Panx1 fluorescence was measured subtracting the mean fluorescence 1 μm under the cell periphery to the total cell fluorescence intensity. Pearson’s correlation was employed to determine the level of colocalization between Panx1 and biotin.

### Amperometric detection of exocytosis in cultured chromaffin cells

Amperometric recordings were performed as previously described (Ardiles et al., 2006). Carbon fiber electrodes (5 μm diameter, Thornel P-55; Amoco Performance Product, Greenville, USA) held at a potential of 650 mV were used to detect single exocytotic events. A HEKA EPC10 amplifier (HEKA Elektronik, Lambrecht/Pfalz, Germany) controlled by the PatchMaster software (HEKA Elektronik) allowed us to obtain the amperometric signals, which were low-pass filtered at 1 kHz and digitized at 5 Hz. During the recording, cells were maintained in Kreb’s-Hepes solution. The exocytosis was evoked by a 10 s pressure ejection of 50 μM DMPP. In order to evaluate the role of Panx1 channels in exocytosis, cells were pre-incubated for 15 min with Cbx (5 μM), or ^10^Panx1 or ^10^Panx1 scrb peptides (200 μM) at 37°C. These reagents were maintained in the bath solution during the entire recording. Single exocytotic events were analyzed using a written macro for IGOR (Wavemetrics), obtained from Dr. R. Borges.^1^ Only spikes with Imax > 5SD of the noise were counted and analyzed.

### Cytosolic [Ca^2+^] measurements

Variations of cytosolic [Ca^2+^] were determined using microfluorometry (Cárdenas et al., 2002). Chromaffin cells cultured on glass coverslips were incubated for 40 min at 37°C with 5 mM Indo-1 AM (Molecular Probes, Life technologies) in 0.1% pluronic acid and then washed with Kreb’s-Hepes solution (pH 7.4). The coverslips were mounted in a perfusion chamber and placed on the stage of a fluorescence-inverted microscope (Diaphot- 200, Nikon Corp. Tokyo, Japan). The microscope was equipped with two dichroic mirrors: the first one sent excitation light (355 nm) to the cell and the second mirror split the fluorescent light emitted by intracellular Indo-1 (>400 nm) into beams of light centered at 410 and 485 nm. The intensity of the light at each wavelength was measured continuously using two photomultipliers and the digital signal was obtained using HEKA EPC10 amplifier (HEKA Elektronik) controlled by the PatchMaster software (HEKA Elektronik). The cytosolic [Ca^2+^] was calculated from the F_410_/F_485_ ratio using the following formula: [Ca^2+^] = K_d_ (R−R_min_)/(R_max_/R). The Ca^2+^ dissociation constant for Indo-1 AM, K_d_, was obtained with a calibration curve using various known [Ca^2+^]. R_min_ was determined incubating the cells with EGTA (10 mM) and R_max_ with a solution containing 10 μM ionomycin with 10 mM CaCl_2_. Cells were incubated at 37°C with Cbx (5 μM), probenecid (200 μM), ^10^Panx1 (200 μM) or ^10^Panx1 scrb peptide (200 μM) 15 min before the experiments. These inhibitors were maintained in the bath solution during the entire recording. The Ca^2+^ signals were evoked by a 10 s pressure ejection of 50 μM DMPP or 50 mM caffeine. In experiments done with DMPP, the cells were kept in the Kreb’s-Hepes solution, but the stimulation with caffeine was done in a Ca^2+^-free Kreb’s-Hepes solution (in mM: 140 NaCl, 5.9 KCl, 1.2 MgCl_2_, 2 EGTA, 10 Hepes-NaOH, 10 glucose).

### DAPI uptake

Chromaffin cells cultured on 12 mm coverslips were washed twice with Krebś Hepes solution and incubated for 15 min in Kreb’s Hepes solution with or without Cbx (5 μM) or probenecid (200 μM) at 37°C. Then, the cells were incubated for 10 s with a Kreb’s Hepes solution containing 50 μM DAPI (Sigma) in the absence or presence of 50 μM DMPP and the respective Panx1 inhibitors. Then the solution was replaced the same Kreb’s Hepes solutions without DMPP for 2 min and the cells were fixed with 4% PFA in PBS for 15 min at 4°C. Finally, coverslips were washed 3 times and mounted with DAKO fluorescent mounting medium (Dako, Glostrup, Denmark). Stained cells were visualized with a Nikon C1 Plus laser-scanning confocal microscope, equipped with a 40X objective (NA = 1.30) and excited with laser lines of 408. Confocal acquisitions were analyzed and processed using Image-J software (NIH, USA). Three independent background fluorescence intensity measurements were averaged and subtracted from the fluorescence intensity of each cell. DAPI uptake, expressed in AU/μm^2^, was evaluated calculating the fluorescence intensity of each chromaffin cell nucleus, divided by the nucleus area.

#### Data analysis and statistics

Data of catecholamine secretion from adrenal glands correspond to the mean ± SEM of 9 to 10 glands. Amperometric spikes, cytosolic [Ca^2+^] and DAPI uptake were averaged by individual cells, where “*n*” refers to the number of tested cells. Data presented correspond to means ± SEM of cell averages from at least three different cultures. The statistical significance of the differences was evaluated using Krukal-Wallis test for nonparametric data, level of *p* < 0.05 was considered statistically significant (*).

#### Ethics statement

The present work includes the use of bovine adrenal glands obtained from a local slaughterhouse, Frigorific Don Pedro, certificated (Livestock role 04.2.03.0002) by the Agriculture and Livestock Service of the Chilean Government. The slaughterhouse is regularly inspected by a veterinarian of the Chilean Health Service. Transport, processing and elimination of the samples were carried out in strict accordance with the Article 86 of the Sanitary Regulations of the Chilean Government (Supreme decree Nu 977/96). Panx1 knock-out (KO) C57BL/6 mice previously described by Bargiotas et al. (2011) were kindly provided by Dr. Hannah Monyer, University Heidelberg, Germany. These animals were bred in the Animal Facilities of the Pontifícia Universidad Catόlica de Chile. Wild type C57BL/6 mice were used as control. The use of KO mice was limited to crucial experiments to reduce the number of animals sacrificed. Mouse brain extract were obtained using 9–12 months old male.

All the protocols described in this article were approved by a Committee of Bioethics and Biosafety of the Faculty of Science, University of Valparaíso, directed by Professor Juan Carlos Espinoza, on May, 2, 2011.

## Results

### Panx1 is expressed in the adrenal gland and participates in the secretory response induced by the activation of nicotinic receptors

**Figure 1:**
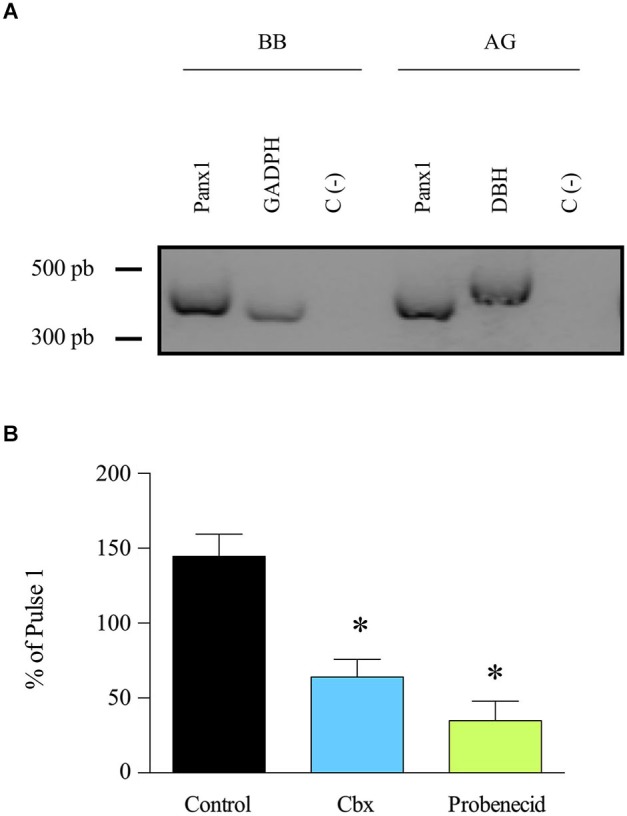
**Panx1 is expressed in adrenal gland and participates in the release of catecholamines**. **(A)** Expression of Panx1 was evaluated by RT-PCR using Panx1 primers. The RT-PCR was performed on mRNA extracted from bovine brain (BB) (positive control) and adrenal gland (AG). GADPH and DBH were used as amplification controls; they lead to the generation of 334 pb and 480 pb transcripts, respectively. A negative control without template was also included (C-). The presence of Panx1 transcript (396 pb) was detected in bovine brain as well as in adrenal gland. **(B)** Entire adrenal glands were perfused with Kreb’s solution for 1 h, and then the release of catecholamines was induced by a 2 min pulse of the nicotinic agonist DMPP (50 μM). Fifteen minutes before the second pulse, the glands were treated with Cbx (5 μM) or probenecid (200 μM). The drugs were maintained in the perfused solution during the entire sample recollection. Absorbance of the catecholamine oxidation products was measured at 540 nm. Data, expressed as the percentage of catecholamines secreted during the second pulse with respect to the first pulse, are means ± SEM of control glands (*n* = 10), or glands treated with carbenoxolone (Cbx) (*n* = 9) or probenecid (*n* = 10). * *p* < 0.05 compared with control glands (Krukal-Wallis test).

**Figure 2:**
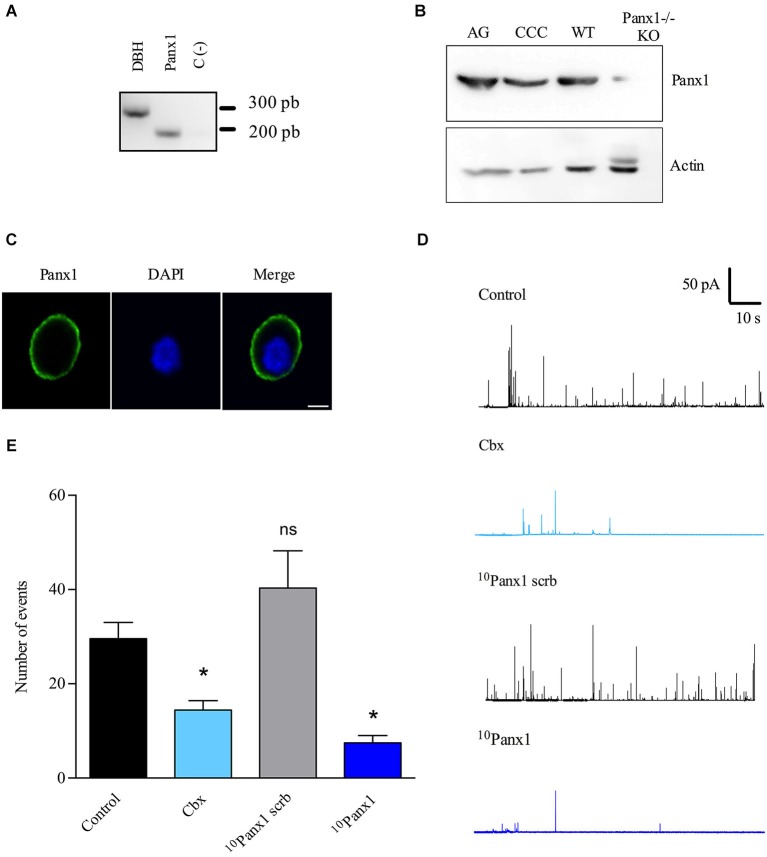
**Panx1 is expressed in cultured chromaffin cells and contributes to the exocytotic release of catecholamines. (A)** A single cell RT-PCR assay was performed on single chromaffin cell using a nested PCR procedure in order to detect the presence of Panx1 mRNA. After the second PCR, the predicted transcript sizes were observed at 318 pb for Panx1 and 248 pb for DBH (positive control), thus confirming the expression of Panx1 in cultured bovine chromaffin cells. **(B)** Expression of Panx1 at the protein level. Protein extract (40 μg) from adrenal gland (AG) or cultured chromaffin cells (CCC) were used for electrophoresis and western blot analysis using a specific serum against Panx1. A band around 50 kDa was detected confirming the expression of Panx1 in both samples. Brain extracts from WT or Panx1^−/−^ KO mice were used as positive and negative control. Anti-actin antibody was used as a loaded control. **(C)** Localization of Panx1 in cultured chromaffin cells. Confocal immunofluorescence images were obtained by labeling chromaffin cells with a polyclonal anti-Panx1 serum and visualized with an α-CY2-conjugated secondary antibody. Nuclei were visualized with DAPI staining. Scale bar = 10 μm. **(D,E)** The secretory activity was evoked by a 10 s pulse of the nicotinic agonist DMPP (50 μM) and monitored by amperometry. Cells were pre-incubated with the different inhibitors during 15 min at 37°C, which were maintained in the bath solution during the whole experiment. **(D)** Representative amperometry traces from control cells or cells treated with Cbx (5 μM), ^10^Panx1 scrb peptide (200 μM) or ^10^Panx1 peptide (200 μM). Note that Panx1 channel inhibitors decreased the number of exocytotic events. **(E)** Data show average values ± SEM of the number of events during 100 s recording of control cells (*n* = 55), or cells treated with Cbx (*n* = 20), ^10^Panx1 scrb peptide (*n* = 18) or ^10^Panx1 peptide (*n* = 25) from three to five different cultures. ns = non-significant, * *p* < 0.05 (Krukal-Wallis test) compre with control cells.

Panx1 is expressed in various tissues including neuroendocrine tissues such as the pituitary gland (Li et al., 2011) but until now, its expression in the adrenal gland remains unknown. To investigate Panx1 expression in this tissue, we performed an RT-PCR assay of total RNA obtained from bovine adrenal glands. Bovine brain RNA was used as a positive control. Panx1 transcripts were detected in both tissues (Figure 1A). The expression of the protein in the adrenal gland was confirmed by western blot using a specific polyclonal serum against Panx1 (Figure 2B). Next, we studied the possible implication of Panx1 expression in the release of catecholamine from intact adrenal glands. To this end, we used two different Panx1 channel inhibitors: Cbx, which at 5 μM blocks Panx1 channels, but not connexin based channels (Bruzzone et al., 2005), and probenecid (200 μM), a Panx1 channel inhibitor (Silverman et al., 2008). To mimic the physiological condition, the glands were stimulated with the nicotinic agonist DMPP. First, the glands were perfused with Krebs’s solution for 1 h, then the secretory activity was induced with two 2 min pulses of the nicotinic agonist DMPP (50 μM) applied every 45 min. A group of glands was treated with probenecid or Cbx 15 min before and during the second pulse. In these experiments, the first pulse was used as an internal control. Figure 1B shows the catecholamine release after the second DMPP pulse expressed as a percentage of the release induced by the first pulse. In control glands, the secretory response increased up to 144.6% after the second stimulation. Conversely, the treatment with Cbx or probenecid significantly decreased secretory activity of the adrenal gland to 64.1% and 34.9%, respectively. Taken together, these results demonstrate that Panx1 channels regulate the secretory activity of the adrenal gland. Therefore, we decided to study the involved mechanism in cultured chromaffin cells.

### Panx1 is expressed in cultured chromaffin cells and controls the number of secretory events

We first checked that Panx1 mRNA is expressed in chromaffin cells using single cell RT-PCR. To do so, the cytoplasm of a single chromaffin cell was extracted and we performed the RT-PCR with a nested PCR procedure by using two pairs of primers designed for Panx1. DBH mRNA was used as a positive control. In this assay, we also observed the presence of Panx1 mRNA (Figure 2A), confirming its expression in chromaffin cells. In order to verify that Panx1 is expressed at the protein level and how it is distributed in cultured chromaffin cells, we performed western blot and immunofluorescence assays. By western blot, we detected the presence of a band of around 50 kDa in extracts from cultured chromaffin cells and adrenal glands (Figure 2B). Brain extracts from WT or Panx1^−/−^ mice were used as positive control and negative control, respectively. An anti-actin antibody was used as a loaded control. Samples processed for immunofluorescence were visualized using a confocal microscope and we found that cultured chromaffin cells were positively immunolabeled by the anti-Panx1 serum. Panx1 was essentially found at the cell periphery (Figure 2C), suggesting that it is mainly localized at the plasma membrane of the chromaffin cells.

Then, we monitored exocytosis in chromaffin cells using amperometry, since this technique provides information about the characteristics of individual release events (González-Jamett et al., 2010). The exocytosis was induced with 50 μM DMPP and Panx1 channels were inhibited with 5 μM Cbx or the mimic peptide ^10^Panx1 (200 μM), which is a specific Panx1 channel blocker (Pelegrin and Surprenant, 2006). Each inhibitor was added to the culture medium 15 min before the experiment and maintained in the bath solution during the entire recording. The scramble ^10^Panx1 peptide (^10^Panx1 scrb) was used as a negative control. All the experiments were performed on uncoupled cells to rule out any possible implication of gap junctional coupling with neighboring cells. Figure 2D shows representative amperometric traces of each condition. In non-treated cells (control), a 10 s pulse with 50 μM DMPP induced 29.6 ± 3.4 amperometric spikes in 100 s (*n* = 57). The number of release events was not significantly affected by ^10^Panx1 scrb (40.3 ± 7.8). However, it was significantly diminished by Cbx or ^10^Panx1, as compared with non-treated cells (control) or cells treated with the ^10^Panx1 scrb, respectively (Figure 2E). ^10^Panx1 and Cbx reduced the event number by 81.4% and 51.3% respectively.

### Panx1 channels regulate the kinetic of the single release events

**Figure 3:**
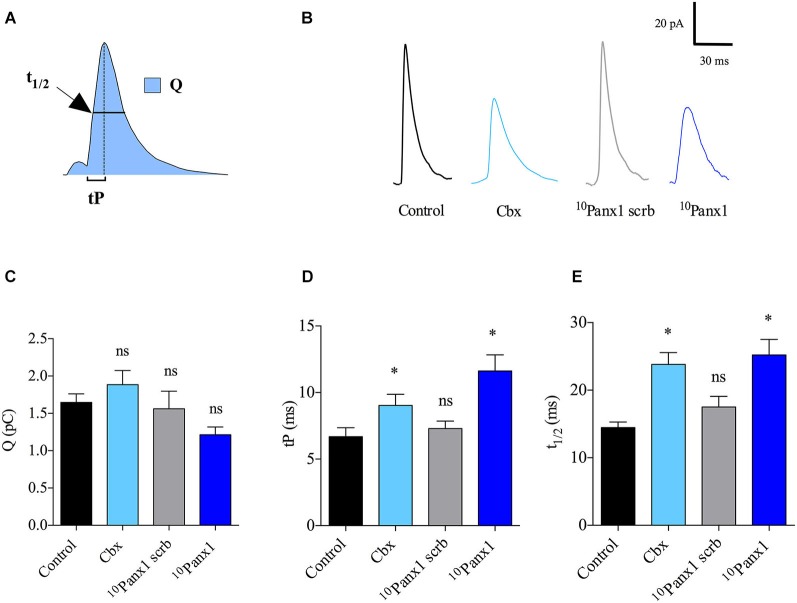
**Panx1 channels regulate the kinetics of single exocytotic events. (A)** Schema of an amperometric spike with the different parameters analyzed: quantal size (Q), time to peak (tP) and half width (t_1/2_). **(B)** Representative amperometric spikes from control cells or cells treated with 5 μM Cbx, 200 μM ^10^Panx1 scrb peptide (*n* = 18) or 200 μM ^10^Panx1 peptide (*n* = 25). **(C–E)** Data show average values of Q, tP and t_1/2_ ± SEM from control cells (*n* = 55), or cells treated with 5 μM Cbx (*n* = 20), 200 μM ^10^Panx1 scrb peptide (*n* = 18) or 200 μM ^10^Panx1 peptide (*n* = 25) from three to five different cultures. Parameter values correspond to the mean values of the events from individual cells, thus “n” corresponds to the number of cells analyzed in each group. * *p* < 0.05 (Mann-Whitney test) compare with control cells. Note that treatment of chromaffin cells with the Panx1 channel inhibitors did not affect Q **(C)**, but slows down the kinetics of single exocytotic events, as observed by an increase of tP **(D)** and t_1/2_ **(E)**. ns = non-significant, * *p* < 0.05 (Krukal-Wallis test).

In order to understand how Panx1 channels affect the characteristics of individual release events, we analyzed for each amperometric spike the quantal size (Q), which is proportional to the amount of catecholamines released per event, the time to peak (tP) or rising time that reflects the speed of the expansion of the fusion pore, and the half-width (t_1/2_) that reflects the duration of the exocytotic events (Neco et al., 2008; Figure 3A). Figure 3B shows examples of amperometric spike of control cells or cells treated with the different Panx1 inhibitors. Q, tP and t_1/2_ values in control cells stimulated with 50 μM DMPP were 1.6 ± 0.1 pC, 6.6 ± 0.6 ms and 14.5 ± 0.8 ms, respectively. As compared with control cells, the cell treatment with ^10^Panx1 scrb did not affect any of the amperometric parameters. In this last condition Q, tP and t_1/2_ values were 1.6 ± 0.2 pC, 7.3 ± 0.5 ms and 14.4 ± 0.8 ms, respectively. The treatment with Cbx or ^10^Panx1 also did not significantly change Q values compared with control cells or cells treated with ^10^Panx1 (Figure 3C). However, in the presence of these Panx1 channel inhibitors, tP values were significantly higher (11.6 ± 1.2 and 9.0 ± 0.8 ms for Cbx or ^10^Panx1 treatment, respectively) (Figure 3D). As shown in Figure 3E, a similar trend was observed for t_1/2_ values (25.2 ± 2.3 and 23.8 ± 1.7 ms for Cbx or ^10^Panx1 treatment, respectively). These results indicate that Panx1 channels control the number of release events as well as the kinetics of single fusion events.

### Panx1 channels contribute to the Ca^2+^ signals induced by activation of nicotinic receptors

**Figure 4:**
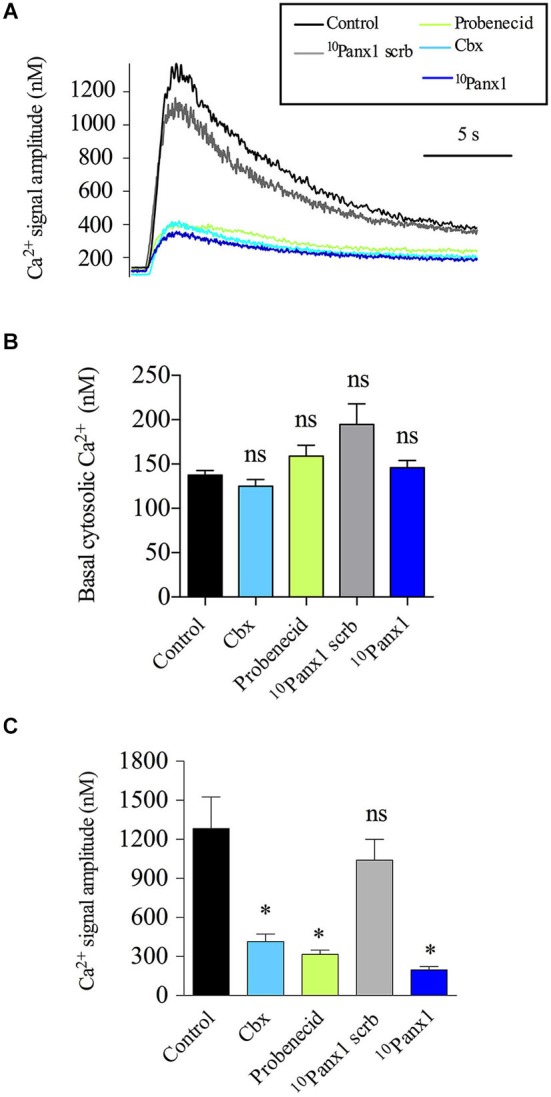
**Panx1 channels contribute to the Ca^2+^ signal induced by the activation of nicotinic receptors**. Indo-1 loaded chromaffin cells were stimulated with a 10 s pulse of the nicotinic agonist DMPP (50 μM), and the cytosolic [Ca^2+^] was monitored by microfluorometry. **(A)** Representative traces of [Ca^2+^] rises induced by DMPP. Note that the evoked [Ca^2+^] rise was reduced in the presence of Cbx (5 μM), probenecid (200 μM) or ^10^Panx1 peptide (200 μM). **(B,C)** Data show means ± SEM of basal cytosolic [Ca^2+^] **(B)** or the maximum amplitude of the Ca^2+^ signals induced by DMPP **(C)** in control cells (*n* = 69), cells treated with probenecid (*n* = 40), ^10^Panx1 scrb peptide (*n* = 37) or ^10^Panx1 peptide (*n* = 31). Cbx, probenecid and ^10^Panx1 peptide treatment significantly reduced the amplitude of the DMPP-induced Ca^2+^ signal. ns = non-significant, * *p* < 0.05 (Krukal-Wallis test) compare with control cells.

Given that the secretory response in chromaffin cells is triggered by a transient increase in the cytosolic [Ca^2+^] and that Panx1 channels have been implicated in the regulation of Ca^2+^ signals (Vanden Abeele et al., 2006; Pinheiro et al., 2013), we explored the possibility that Panx1 channels affect the secretory activity in chromaffin cells by contributing to the Ca^2+^ signal induced by the activation of nicotinic receptors. Thus, we analyzed the impact of different Panx1 channel inhibitors on the Ca^2+^ signals evoked by the nicotinic agonist DMPP in cells loaded with the Ca^2+^ probe Indo-1. Figure 4A shows representative traces of cytosolic [Ca^2+^] signals in cells treated with the different inhibitors. In control cells, the cytosolic [Ca^2+^] in resting condition was 137.5 ± 5.1 nM, cell stimulation with a 10 s pulse of 50 μM DMPP led to a transient increase of the cytosolic [Ca^2+^] with an amplitude of 1.2 ± 0.2 μM (Figures 4B,C). As shown in Figure 4B, none of the different Panx1 channel inhibitors affected the resting cytosolic [Ca^2+^] (125.1 ± 7.4 nM, 194.7 ± 23.1 nM, 145.8 ± 8.1 nM and 145.8 ± 8.1 nM for cells treated with Cbx, probenecid, ^10^Panx1 scrb and ^10^Panx1, respectively). However, treatment with Cbx, probenecid or ^10^Panx1 significantly reduced the amplitude of the Ca^2+^ signal induced by DMPP by ~68%, ~75% and ~85%, respectively, compared to control cells (Figure 4C). As we found for the secretory response, the strongest inhibition was observed after treatment with ^10^Panx1.

### Panx1 channels expressed at the plasma membrane contribute to the amplification of DMPP-induced Ca^2+^ signals

**Figure 5:**
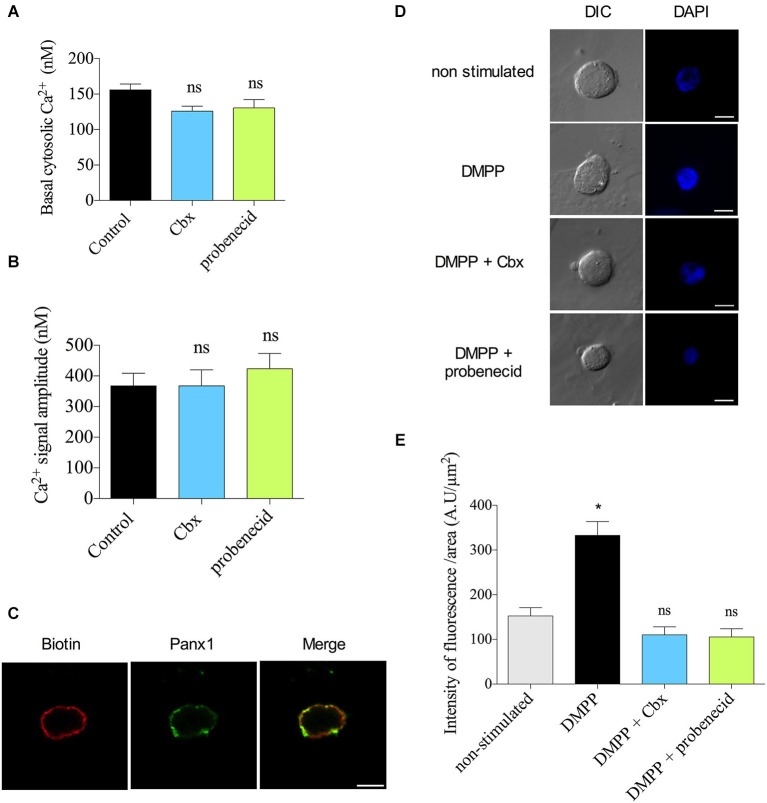
**The Panx1 channels do not contribute to the Ca^2+^ signal induced by caffeine but form functional channels at the plasma membrane. (A,B)** Indo-1 loaded chromaffin cells were maintained in a Ca^2+^-free Kreb’s Hepes solution (in mM: 140 NaCl, 5.9 KCl, 1.2 MgCl_2_, 2 EGTA, 10 Hepes-NaOH, 10 glucose) and stimulated with a 10 s pulse of 50 mM caffeine. The cytosolic [Ca^2+^] was monitored by microfluorometry. Data show means ± SEM of basal cytosolic [Ca^2+^] **(A)** or the maximum amplitude of the Ca^2+^ signals induced by caffeine **(B)** in control cells (*n* = 22), or cells treated with probenecid (*n* = 22) or Cbx (*n* = 21). ns = non-significant, * *p* < 0.05 (Krukal-Wallis test). **(C)** Colocalization of Panx1 with extracellular biotin at plasma membrane. Confocal immunofluorescence images were obtained by labeling external plasma membrane protein of chromaffin cells with biotin, and Panx1 with a polyclonal anti-Panx1 serum. Biotin labeling was visualized with avidin Cy3 and Panx1 with α-CY2-conjugated secondary antibody. Nuclei were visualized with DAPI staining. Scale bar = 10 μm. **(D,E)** DAPI uptake experiments were performed incubating the cells for 10 s in a Kreb’s HEPES solution containing 50 μM DAPI without (unstimulated cells) or with nicotinic agonist receptor DMPP (50 μM). Then cells were incubated with Kreb’s HEPES solution containing 50 μM DAPI for 2 min and immediately fixed with 4% PFA (in PBS, pH 7.4). After 3 washes with PBS (pH 7.4), coverslip were mounted for visualization. Treatment with probenecid (200 μM) Cbx (5 μM) were performed 15 min before the experiments and the inhibitors were maintained in the bath solution until cell fixation. **(D)** Confocal imaging and **(E)** quantification of DAPI uptake in unstimulated (*n* = 24) or DMPP stimulated cells, nontreated (*n* = 21) or treated with the Panx channel inhibitors (*n* = 24 and *n* = 19 for probenecid and Cbx respectively). Scale bar = 10 μm. Data shows mean ± SEM of nucleus fluorescence intensity per nucleus area (AU/μm^2^). ns = non-significant, * *p* < 0.05 (Krukal-Wallis test) compare with non-stimulated cells.

Finally, we explored the mechanism by which Panx1 contributes to the Ca^2+^ response to nicotinic receptor stimulation. One possibility is that the Ca^2+^ signal is mediated by Panx1 channels present at the ER membrane. This idea was supported by two facts: (1) the Ca^2+^ release from ER importantly contributes to the increase of the [Ca^2+^] in response to nicotinic receptor stimulation (del Barrio et al., 2011); and (2) in other cell types, Panx1 channels present at ER mediate the Ca^2+^ release from intracellular stores (D’hondt et al., 2011). Therefore, we evaluated the effect of Panx1 inhibitors on the Ca^2+^ release from the ER induced by 50 mM caffeine (Cheek et al., 1993; Alonso et al., 1999). These experiments were done in a Ca^2+^-free medium. Under these conditions, treatment with Cbx (5 μM) or probenecid (200 μM) did not affect the basal [Ca^2+^] (Figure 5A) or the amplitude of the Ca^2+^ signal (Figure 5B). Thus, the mechanism by which Panx1 contributed to the Ca^2+^ signals appears to be independent of ER stores.

Another possibility is that Panx1 channels present at the cell periphery contribute to the amplification of the Ca^2+^ response to the nicotinic receptor stimulation. An amplification of Ca^2+^ influx mediated by plasma membrane Panx1 channels has been observed T-cell and platelet (Woehrle et al., 2010; Taylor et al., 2014). Therefore, we evaluated the presence and the functional state of Panx1 channels at the plasma membrane of chromaffin cells. In order to determine if Panx1 is present at this compartment, we co-labeled cells with anti-Panx1 antibody and extracellular biotin, which binds to primary amines of cell surface proteins (Turvy and Blum, 2001). Confocal acquisition showed colocalization of the two markers (Figure 5C). The mean score for the Pearson coefficient between biotin and Panx1 was 0.71 ± 0.02 (*n* = 18), confirming the presence of Panx1 at the plasma membrane. To determine the functional state of plasma membrane Panx1, we performed dye uptake experiments using DAPI. Cultured chromaffin cells were washed and incubated 10 s in a solution containing 50 μM DAPI in the presence (stimulated cells) or absence (non-stimulated cells) of 50 μM DMPP. Then the cells were incubated in 50 μM DAPI in Kreb’s HEPES solution for 2 min and immediately fixed with PFA. Confocal acquisition images and fluorescent analysis showed a low basal DAPI uptake in non-stimulated control cells (152 ± 18 A.U/μm^2^), but the nucleus fluorescence intensity significantly increased in cells stimulated with DMPP (332 ± 31 A.U/μm^2^). Interestingly, cell treatment with 5 μM Cbx or 200 μM probenecid reduced the dye uptake to values comparable to those observed in non-stimulated cells (105 ± 18 and 101 ± 17 A.U/μm^2^ respectively) (Figures 5D,E). Taking together, these results strongly suggest that Panx1 channels present at the plasma membrane contribute to the DMPP-induced Ca^2+^ signal in chromaffin cells.

## Discussion

The present work demonstrates that Panx1 is expressed in bovine adrenal glands and is involved in the regulation of the catecholamine release evoked by nicotinic receptor activation. At the cellular level, we found that Panx1 is expressed at the plasma membrane of cultured chromaffin cells, where it contributes to the exocytotic release process by regulating the Ca^2+^ signal induced by the activation of nicotinic receptors. These findings define Panx1 channels as new actors in the regulation of the catecholamine release and suggest that these channels play a relevant role in the response to stress. As we discuss below, the peculiarities of the regulation of chromaffin cell activity by Panx1 channels enlighten us about the underlying mechanisms.

### Panx1 channels amplify the Ca^2+^ signals in chromaffin cells, impacting the exocytotic release

Until now, most evidence about the nature of the Ca^2+^ signal induced by the activation of nicotinic receptors is explained by Ca^2+^ entry through nicotinic receptors and voltage-dependent Ca^2+^ channels (Arnáiz-Cot et al., 2008). The mechanism of the Ca^2+^ -induced Ca^2+^ -release from the ER is the other important pathway that contributes to the increase of cytosolic Ca^2+^ (del Barrio et al., 2011). As recently demonstrated, depending on the type of nicotinic receptor that is activated, the voltage-dependent Ca^2+^ channels contribute to the Ca^2+^ signals by 15–20%, while the Ca^2+^ -release from the ER contributes over 60% (del Barrio et al., 2011). According to our results using ^10^Panx1, Panx1 channels contribute ~85% to the Ca^2+^ signal induced by the nicotinic agonist DMPP (Figure 4C). Similar results were obtained with the different Panx1 channel inhibitors (Figure 4C). These results are in agreement with the fact that Panx1 importantly regulates both the number of exocytotic events in cultured chromaffin cells (Figures 2D,E) and the global secretion of catecholamines in perfused glands (Figure 1B).

Indeed, the amplitude of the Ca^2+^ signal defines the number of fusion events (Wang et al., 2006; Ardiles et al., 2007). Furthermore, the cytosolic [Ca^2+^] reached at the release sites can also determine the kinetics of the fusion pore expansion; an intermediate structure formed during the exocytosis processes (Lindau and Alvarez de Toledo, 2003). By using amperometry, the latter is reflected in the rise time and the duration of the single release events (Grabner and Fox, 2006; Ardiles et al., 2007). Thus, the slow-down of the rise time (tP) and the lengthening of the event duration (t_1/2_) observed in the presence of the Panx1 channel inhibitors (Figure 3) could be also a consequence of the effect of these agents on the Ca^2+^ signal amplitude. Together these findings reveal that Panx1 channels constitute a new mechanism that importantly contributes to the Ca^2+^ response to the nicotinic receptor activation and impacts the exocytotic release of catecholamines.

### Functional Panx1 channels present at the plasma membrane contribute to the Ca^2+^ signals induce by the activation of nicotinic receptors

As aforementioned, the Ca^2+^ released from the ER in chromaffin cells importantly contributes to the Ca^2+^ signal induced by the activation of nicotinic receptors (del Barrio et al., 2011). On the other hand, the contribution of Panx1 channels to the Ca^2+^ signals could be also mediated by Panx1 channels present at the ER membrane (D’hondt et al., 2011). In prostate cancer cells Panx1 forms Ca^2+^ channels in the ER mediating the Ca^2+^ release from intracellular stores (Vanden Abeele et al., 2006). In osteoblasts, Panx3 also forms channels in the ER that are activated by PI3K–Akt signaling (Ishikawa et al., 2011). However, in our model, the contribution of Panx1 to the signals in chromaffin cells appears to not come from channels present at the ER because Panx1 inhibition did not affect the Ca^2+^ signal induced by caffeine in the absence of extracellular Ca^2+^ (Figure 5B). This idea is also supported by the fact that the extracellular application of ^10^Panx1 (which theoretically does not cross the plasma membrane) decreased the secretory activity of chromaffin cells (Figures 2D,E) and the Ca^2+^ signal induced by DMPP (Figure 4). In addition, the facts that Panx1 was inmunodetected mainly at the plasma membrane (Figure 5C) and a DMPP-induced DAPI uptake was blocked with the Panx inhibitors Cbx and Probenecid (Figures 5D,E), strongly support the idea of functional Panx1 channels at the plasma membrane. Given that Panx1 channels are permeable to Ca^2+^ (Vanden Abeele et al., 2006), a possible mechanism is that the Ca^2+^ entry mediated by Panx1 channels amplifies of the Ca^2+^ signal that induces the secretory process.

### Panx1 channels appear to be activated just during the secretory response

As mentioned in the result section, the blockade of Panx1 channels does not affect the basal [Ca^2+^] (Figure 4B); conversely it decreased the amplitude of the Ca^2+^ signal induced by the nicotinic agonist DMPP. Furthermore, in resting condition, chromaffin cells almost did not uptake DAPI, but the stimulation of nicotinic receptors with DMPP significantly increased the uptake, which is completely block by Cbx- and probenecid (Figures 5E,F). Thus these findings suggest that Panx1 channels are not activated in a resting condition; instead they appear to become functional upon activation of the nicotinic receptor.

Reportedly, Panx1 channels are activated by high extracellular potassium ions (Silverman et al., 2009), membrane depolarization (Dahl and Locovei, 2006), mechanical stimulation (Bao et al., 2004), extracellular ATP through the activation of P2X (Pelegrin and Surprenant, 2006; Woehrle et al., 2010) or P2Y receptors (Locovei et al., 2006) and possibly by Src kinases (Weilinger et al., 2012). Some of mentioned mechanisms are triggered by the stimulation of nicotinic receptors in chromaffin cells, such as membrane depolarization (Pérez-Alvarez et al., 2012), Src kinase activation (Allen et al., 1996) and ATP release (Rojas et al., 1985). Thus, one or several of these events could activate Panx1 channels after the stimulation of nicotinic receptors.

### Is the activation of Panx1 channels in chromaffin cells coupled to purinergic receptors?

Panx1 channels mediate Ca^2+^ influx to the cells through their association with the P2X_7_R (Pelegrin et al., 2008; Iglesias et al., 2009). In this mechanism, the release of ATP through Panx1 channels activates P2X_7_Rs, which in turn transiently increases the cytosolic [Ca^2+^] (see review Baroja-Mazo et al., 2013). Hitherto there is no study showing the expression P2X_7_R in chromaffin cells. However, by using different agonists and antagonists, Tomé et al. (2007b) suggest the presence of functional purinergic P2X_7_R receptor, but only in a fraction (app. 20%) of the cultured bovine chromaffin cells.

Panx1 channels could also be functionally associated with P2Y_1_ and P2Y_2_ receptors (Locovei et al., 2006), and chromaffin cells expressed P2Y receptors that mediate Ca^2+^ signals (Tomé et al., 2007a,b). However, and as observed with P2X receptors, they are functionally expressed in only a small fraction of cultured bovine chromaffin cells (Tomé et al., 2007a,b). On the other hand, P2Y receptors in chromaffin cells also inhibit the activity of voltage-dependent Ca^2+^ channels (Ennion et al., 2004; Hernández et al., 2011). Therefore, the dual action of ATP in chromaffin cells makes its actions on the Ca^2+^ signals complex (Reichsman et al., 1995; Carabelli et al., 2001) and therefore their function and association to Panx1 channels needs further investigation in chromaffin cells.

## Conclusion

Taken together our results reveal a new mechanism that regulates the release of hormones by the adrenal chromaffin cells. In this mechanism the opening of Panx1 channels at the plasma membrane after the activation of nicotinic receptors contributes to the Ca^2+^ signal that triggers exocytosis, resulting in a robust and fast secretory response. This mechanism could have physiological implications during the response to stress.

## Conflict of interest statement

The reviewer Dr. Retamal declares that, despite having collaborated with the authors, the review process was handled objectively. The authors declare that the research was conducted in the absence of any commercial or financial relationships that could be construed as a potential conflict of interest.

## Footnotes

^1^http://rborges.webs.ull.es/index.html
